# Examining the Relationship Between Environmental Factors and Inpatient Hospital Falls: Protocol for a Mixed Methods Study

**DOI:** 10.2196/24974

**Published:** 2021-07-13

**Authors:** Ronald I Shorr, Sherry Ahrentzen, Stephen L Luther, Chad Radwan, Bridget Hahm, Mahshad Kazemzadeh, Slande Alliance, Gail Powell-Cope, Gary M Fischer

**Affiliations:** 1 Geriatric Research Education and Clinical Centers North Florida/South Georgia Veterans Health System Gainesville, FL United States; 2 Shimberg Center for Housing Studies College of Design, Construction and Planning University of Florida Gainesville, FL United States; 3 Research Service James A Haley Veterans Hospital Tampa, FL United States; 4 Research Service North Florida/South Georgia Veterans Health System Gainesville, FL United States; 5 Office of Facilities Standards Service/Office of Facilities Planning Office of Construction and Facilities Management Department of Veterans Affairs Washington, DC United States

**Keywords:** falls, accidental falls, hospital design and construction, health facility environment, hospital units, evidence-based facility design, nursing, environmental factors, well-being, accident

## Abstract

**Background:**

Patient falls are the most common adverse events reported in hospitals. Although it is well understood that the physical hospital environment contributes to nearly 40% of severe or fatal hospital falls, there are significant gaps in the knowledge about the relationship between inpatient unit design and fall rates. The few studies that have examined unit design have been conducted in a single hospital (non-Veterans Health Administration [VHA]) or a small number of inpatient units, limiting generalizability. The goal of this study is to identify unit design factors contributing to inpatient falls in the VHA.

**Objective:**

The first aim of the study is to investigate frontline and management perceptions of and experiences with veteran falls as they pertain to inpatient environmental factors. An iterative rapid assessment process will be used to analyze the data. Interview findings will directly inform the development of an environmental assessment survey to be conducted as part of aim 2 and to contribute to interpretation of aim 2. The second aim of this study is to quantify unit design factors and compare spatial and environmental factors of units with higher- versus lower-than-expected fall rates.

**Methods:**

We will first conduct walk-through interviews with facility personnel in 10 medical/surgical units at 3 VHA medical centers to identify environmental fall risk factors. Data will be used to finalize an environmental assessment survey for nurse managers and facilities managers. We will then use fall data from the VA Inpatient Evaluation Center and patient data from additional sources to identify 50 medical/surgical nursing units with higher- and lower-than-expected fall rates. We will measure spatial factors by analyzing computer-aided design files of unit floorplans and environmental factors from the environmental assessment survey. Statistical tests will be performed to identify design factors that distinguish high and low outliers.

**Results:**

The VA Health Services Research and Development Service approved funding for the study. The research protocol was approved by institutional review boards and VA research committees at both sites. Data collection started in February 2018. Results of the data analysis are expected by February 2022. Data collection and analysis was completed for aim 1 with a manuscript of results in progress. For aim 2, the medical/surgical units were categorized into higher- and lower-than-expected fall categories, the environmental assessment surveys were distributed to facility managers and nurse managers. Data to measure spatial characteristics are being compiled.

**Conclusions:**

To our knowledge, this study is the first to objectively identify spatial risks for falls in hospitals within in a large multihospital system. Findings can contribute to evidence-based design guidelines for hospitals such as those of the Facility Guidelines Institute and the Department of Veterans Affairs. The metrics for characterizing spatial features are quantitative indices that could be incorporated in larger scale contextual studies examining contributors to falls, which to date often exclude physical environmental factors at the unit level. Space syntax measures could be used as physical environmental factors in future research examining a range of contextual factors—social, personal, organizational, and environmental—that contribute to patient falls.

**International Registered Report Identifier (IRRID):**

DERR1-10.2196/24974

## Introduction

### Background

According to the Joint Commission, patient falls are hospital-acquired conditions, and falls with serious injury were the most reported sentinel event in 2018 [1]. Inpatient falls result in a financial burden to US medical organizations, contributing to a 61% increase in patient care costs [2] and decreased quality of life for the injured patients. In 2015, the total medical costs for falls totaled more than $50 billion, and Medicare and Medicaid shouldered 75% of these costs [3]. Thus, decreasing the rate of patient falls is a main focus for improving the quality of health care. According to the Joint Commission, physical environment was a common factor contributing to a fall with injury [4]. Although programs that address multiple risk factors have been implemented to reduce hospital falls, none of the commonly used fall risk assessment tools include built environment factors [5]. Retrospective research studies examining the association between the physical environment of the hospital unit and patient falls are few [6], and many of these rely on Likert-type scales of perceived intensity (eg, excellent to poor lighting or sight lines) rather than physical measurement of environmental factors (eg, lumens, distance in linear feet). Many of these studies narrowly focus on design factors immediately impinging on the patient (eg, bedrails) [7] and overlook how physical design affects workflow and care delivery, which in turn affects patient outcomes such as falls [5,8].

Factors in the facility environment that affect social organization and behavior include spatial organization, environmental systems, communication cues, ambient qualities, and architectural details [9]. Of these, spatial organization factors play a prominent role in supporting or hindering behaviors of staff and patients in health care settings. Corridor layout, the location of nursing stations, and placement of interior doors and windows can facilitate or impede staff oversight of patient movement and activities. In one hospital case study, Choi [6] examined the locations of patient falls in relation to physical accessibility and visibility from nurse to patient bed using key locations (nursing station, medications, and a corridor’s circulation path). Using digitized floor plans and spatial analysis software, Choi numerically and graphically analyzed physical accessibility and visibility (ie, sight lines) from key locations to inside patients’ rooms. Findings showed that patient bed visibility from decentralized nurses’ stations and from corridors was significantly associated with the incidence of patient falls. As part of our mixed methods study, we propose a similar approach to physically measure environmental design factors within Department of Veterans Affairs (VA) hospitals that may contribute to patient falls via the manner in which design affects accessibility and visibility. It should be noted that this does not include video monitoring as it may not be not allowed in medical/surgical units. The physical design of a unit can facilitate or impede nursing care, affecting efficiency, stress, and patient care [10]. Hospital unit design experts often ask if the design is (1) flexible, allowing for changes over time, (2) efficient, allowing for quick and easy access to patients, families, computers, and supplies, (3) permitting the most optimal use of the space while increasing unimpeded sight lines, and (4) welcoming to patients and their families.

Data on falls and fall-related injuries have been systematically collected and analyzed for all nonfederal hospital units since 2011. Although the complementary data sources exist for Veterans Health Administration (VHA) hospitals, there are only a few published studies describing the system-wide burden of hospital falls in the VHA system of care [11,12]. In the past decade, hospital and health care design is increasingly guided by evidence-based design and rigorous research to link the spatial design and built characteristics of hospitals to health care outcomes [5,13].

### Study Objectives

This mixed methods study will explore the relationship between unit design and patient falls in VHA medical/surgical units. The goal of aim 1 is to investigate frontline staff and management perceptions of and experiences with veteran falls as they pertain to inpatient environmental factors including patient rooms, bathrooms, nurses’ stations, and hallway design features. We will conduct walk-through interviews in medical/surgical units at 3 VHA medical centers with unit and facility personnel to identify environment-related fall risk factors for patient falls. Interview findings will be used to develop an environmental assessment survey to be conducted as part of aim 2. The goal of aim 2 is to quantify unit design factors and compare spatial and environmental factors of units with higher- versus lower-than-expected fall rates. We will use fall data from the VA Inpatient Evaluation Center and patient data from additional data sources to identify units with higher- and lower-than-expected risk-adjusted fall rates. Predicted values for hospital fall rates will be based on a regression model by using the SAS Proc Mixed procedure employing residual (restricted) maximum likelihood covariance structure and accounting for nesting within Veterans Integrated Service Networks (VHA region) and facility [14]. Differences between predicted and observed values will be based on studentized residuals. We will measure spatial factors by analyzing computer-aided design (AutoCAD) files of unit floorplans and environmental factors based on the environmental assessment survey.

## Methods

### Design

We propose a mixed methods descriptive study to explore the relationship between unit design and patient falls in VHA medical/surgical units. We will use a qualitative approach of participant observation with walk-through interviews to investigate staff and management perceptions of environmental factors that contribute to patient falls (aim 1). We will use quantitative approaches to identify medical/surgical units with higher- or lower-than-expected fall rates and identify spatial and environmental factors that distinguish them (aim 2).

**Figure 1:**
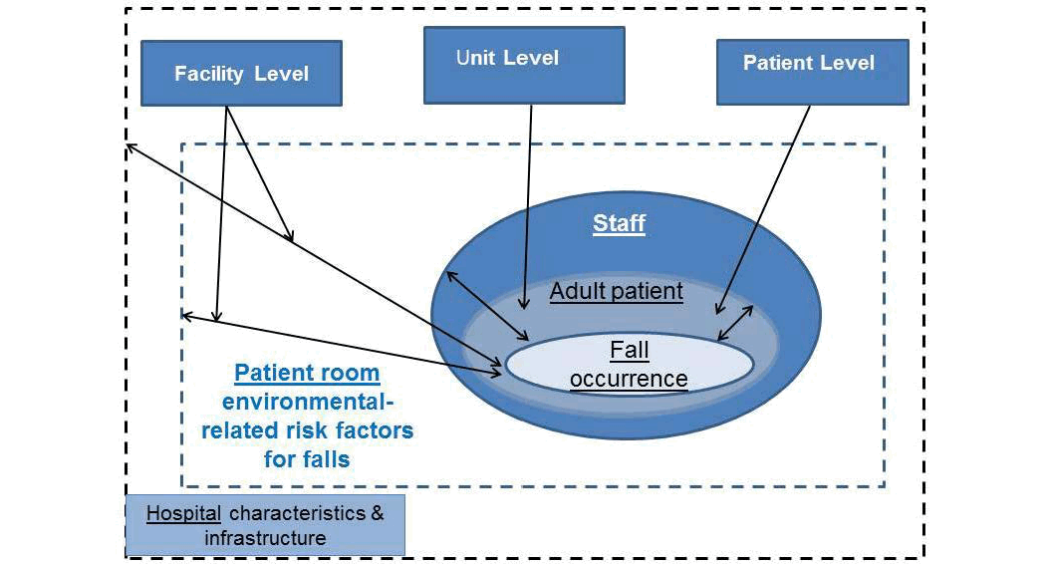
Conceptual model.

The study conceptual framework is based on the box model proposed by Tzeng [15], which acknowledges that fall prevention encompasses patient-, unit-, and facility-level system risk factors (Figure 1). Patient-level characteristics include factors like age, gender, severity of illness/comorbidities, surgical procedures, psychotropic drugs, and altered mental status. Unit-level characteristics include nurse staffing, type of additional unit level and patient room characteristics such as unit accessibility, unit visibility, corridor path length, distance from bed to bathroom, and patient room type. Facility-level characteristics include facility complexity and geographic location.

### Setting and Study Population

The VA is the largest integrated health care system in the United States, consisting of 150 medical centers, nearly 1400 community-based outpatient clinics, community living centers, Vet Centers and domiciliaries. Together, these health care facilities and the more than 53,000 independent licensed health care practitioners who work in them provide comprehensive care to more than 9 million veterans each year [16].

### Inclusion Criteria

Within each facility, personnel at 4 different organizational levels and in different roles will be invited to participate. Participants will be nurse managers, staff nurses, patient care technicians, occupational and physical therapists, and Patient Safety Committee members currently working in medical/surgical units at the facilities. Participants should have at least 1 year of hospital experience at the hospital where they are currently employed. Both day and night shift staff will be recruited.

From all medical/surgical units in the VHA system, we will identify nursing units reporting unusually high and low patient fall rates over time (ie, nursing units showing highest deviation from the rates predicted by an appropriately fitted regression model that includes the known standard predictors of patient falls) [17]. To simplify modeling, we will use aggregated measures of 48 months of observation. Nursing units that report extreme risk-adjusted fall rates may constitute interesting outliers that are not statistical nuisances but which can be used to gain a deeper understanding of the underlying processes leading to patient falls [12].

### Study Procedure Aim 1: Participant Observation

#### Recruitment and Enrollment

We will recruit a purposive [18] sample of clinical staff. We will ask the nursing service administrator at each facility to identify nurse managers of the chosen units and work with the nurse managers to identify individual staff. While identifying staff through administrators and managers may introduce bias, we have used this method successfully in VHA hospitals and have found staff to be forthcoming in responses to interview questions and questionnaires. The unions at each facility endorsed this recruitment method. Once potential interview participants have been identified, project team members will contact each in person or by email or telephone and explain the project. The project manager will explain the informed consent with audio addendum process to each potential participant and, if agreeable, send them the informed consent form. Walk-through interviews will be scheduled in accordance with facility and union standard operating procedures and regulations. We will schedule the interviews at times that are convenient for participants and hospital unit workflow.

#### Interview Procedures

We will conduct 10 walk throughs in 3 facilities yielding 10 group interviews with a total of up to 35 participants. Walk-through interviews are a data gathering technique frequently used in architectural programming to build postoccupancy evaluation. In the walk-through interview, observation and interviewing take place simultaneously. The interviewer walks through designated areas of the building under study with a small group of building occupants. As they walk from area to area, questions are posed by the interviewer, eliciting discussion of key issues, problems, and benefits as perceived by occupants. By coupling the interview with observation of the setting, the walk through triggers detailed recalls of experiences and reactions as related to building factors. This approach allows investigators to triangulate direct observation of the physical factors of the hospital unit with the subjective perspective of the occupant regarding situations associated with it.

The interview team will include (1) a facilitator skilled in qualitative methods who will lead discussions using an interview guide, (2) an assistant facilitator who will take photographs of environmental conditions, and (3) subject matter experts from the study team who will be primarily nonparticipant observers. Interviews will be audiorecorded on password-protected and encryption-capable digital recorders with high-quality audio. Participants will complete an anonymous questionnaire to summarize demographics of the group. At the start of the walk-through interview, the purpose will be explained and any questions from participants will be answered. Participants will be told that their responses will be presented in aggregate or an otherwise deidentified manner and that the interview discussions will remain confidential. Standard response-eliciting techniques will facilitate discussion using generic prompts (eg, “tell me more”), summarizing statements, asking for similar and contrasting opinions, controlling overtalkers, and including all participants in the discussion. The facilitator will use a flexible approach that provides an opportunity to uncover participants’ perspectives using a conversational style.

#### Interview Guide

We developed a semistructured interview guide to elicit responses from all staff participants regarding hospital falls (Multimedia Appendix 1). The interview guide was designed to elicit perceptions and ideas related to unit design factors and their interactions with facility and patient factors that increase the risk of falls. Examples of interview questions include the following:

- What factors at this hospital influence the risk of inpatient falls?
- What efforts are being made at the leadership level to change these factors?
- Do you think unit design has an impact on how staff are able to see patients as they ambulate or get out of their beds?
- As we walk through the unit, please describe where a patient fall occurred and whether you thought the layout of the unit or patient room influenced the fall or its outcome.

#### Data Management

To ensure data quality of walk-through interviews, (1) experienced facilitators will conduct the interviews; (2) data analysis will be concurrent with data collection, and participant feedback will be assessed and compiled; (3) interviews will be audiorecorded to ensure that no information is missed; (4) photographs will be labeled with interview time, location, and corresponding statements referencing design factors of interest; and (5) data analysis will be conducted by one investigator and the qualitative data manager. Additionally, an assistant facilitator will take notes and debrief with the facilitator after the interviews to record shared or unique perspectives. The qualitative data manager will manage data files and organize and assist with data coding. Interview recordings will be reviewed by both facilitators to verify accuracy of field notes and add missing data. Data will be secured and backed up behind the password-protected VA firewall with access limited to team members. Investigators will meet regularly to debrief on data methodologies, data integrity, and data security.

#### Data Analysis Plan

Analysis of interview data will begin following the first interview and will be performed concurrently with ongoing interviews as transcripts are obtained. Insights obtained from continuous analysis will be used to guide further data collection by modifying the interview guide, understanding feedback from participants, and conceptualizing provisional results. A rapid assessment process, an “intensive, team-based, qualitative inquiry using triangulation, iterative data analysis, and additional data collection to quickly develop a preliminary understanding of a situation from the insider’s perspective” [19], will be used to analyze the data. This analysis [20] emphasizes speed of data collection and analysis to focus on programmatic questions or problems encountered by health services researchers. Triangulation of data will be facilitated by including staff who hold different roles, using photographs to validate interviewee responses and collecting data on different units in different hospitals. Interview notes and transcripts will be reviewed by the data team for inductive data reduction to sort, focus, and organize data so themes can be identified and conclusions obtained [20]. Domain names will be developed to correspond with interview questions, and a summary template will be created. Prior to analysis, the template will be internally tested by the team for usability and reliability. Several team members will summarize transcripts and transfer the themes into a matrix to synthesize important findings.

### Study Procedure Aim 2: Modeling Falls Data

#### Procedures

We will obtain the studentized residuals, the difference between the observed data and the predicted value of the observation, based on the model fitted as described below. The top and bottom 25 units (n=50) according to the ranked studentized residuals will be selected to form high and low comparison groups in the second phase. The use of residuals to classify units as high versus low fall rates implies that the amount of unexplained variance is higher or lower than expected after adjusting for available known factors. For example, a unit with the highest predicted adjusted rate may not qualify to be in the unusually high group because it was accurately predicted by the multivariable model; the difference between predicted and observed rates would be relatively small. Alternately, if a medium rate is predicted for a unit but the observed rate is unexpectedly high, this would be regarded as an unusually high value.

To develop the models, we will use the most recent national VHA falls data based on the start date of the study, fiscal years 2013-2017. To provide an estimate of available data for the study, we accessed unit-level fall data for all medical/surgical units through the VHA Support Service Center (317 units and 12,531 unit months). After excluding units with fewer than 3 or more than 200 patient days per month, 11,532 patient months were available for analysis. Multimedia Appendix 2 summarizes the definitions and sources for each of these variables, grouped by model domains.

#### Data Analysis Plan

To develop models, we will investigate the patterns of fall rates per unit per month across 60 months. The degree of variability or stability in rates will guide how to aggregate the falls data. Mean fall rates and risk measure scores will be included in a linear mixed model using SAS (SAS Institute Inc) statistical software. The facility identifier will be treated as a random effect (to adjust for nesting of data within facilities), with all other predictors as fixed effects. We will review regression statistics on the basis of how well the regression model predicts the dependent variable of fall rates.

#### Power Analysis

Statistical testing will be conducted to compare unit design variables between units identified with risk-adjusted high-fall and low-fall rates. Estimated sample sizes (number of nursing units) required to achieve 80% statistical power and the corresponding minimum detectable effect sizes range from a low of 40 (20 high-fall and 20 low-fall) to a high of 50 (25 high-fall and 25 low-fall). In the sample size calculation for the *t* test, the standard deviations were assumed to be unknown and equal. The second sample size calculation was based on a 1-degree of freedom chi-square test. A 2-sided alpha level of .50 was used in both calculations.

### Study Procedure Aim 2: Linking Unit Design and Falls

#### Procedures

To gather data on spatial and environmental characteristics of the units and patient rooms, we will contact facility managers of the sampled hospitals to request AutoCAD digitized documents of floor plans and interior sections of the designated unit and completion of the environmental assessment survey by nurses and facilities management staff developed during aim 1 that includes design features not available on AutoCAD documents (Multimedia Appendix 3). Efforts to maximize cooperation and participation by nurse managers and facilities management will include (1) an email request explaining the study and study findings as highly relevant because they reflect evidence-based design objectives called for by the VA Office of Construction and Facilities Management, (2) request cosigned by the principal investigator and VA Senior Healthcare Architect, (3) follow-up email requests, (4) collaboration with the VHA Office of Nursing Service, and (5) follow-up for nonresponders.

AutoCAD documents are digitized files of floor plans and interior sections of the hospital units. Many spatial features (eg, walls, doors, interior and exterior windows, counter heights) and functional locations (eg, nurses’ stations, medication rooms) are contained in these documents. Project team members will be blinded to whether the floor plans come from high-fall or low-fall units.

#### Spatial Analysis

Space syntax metrics will be computed indirectly by Depthmap software analysis using spatial data entered directly from AutoCAD files. Depthmap is a single software platform that performs spatial network analyses (ie, space syntax) [21,22] using maps of the spatial layout of the hospital unit based on user-designated points within units. Depthmap operates by performing a set of spatial network analyses. One set uses visibility graph analysis [22]. A grid of points is overlaid on a floor plan where each point is connected to every other point that it can see. The visual integration of a point is based on the number of steps it takes to get from that point to any other point within the network. Various graph measures and ratio-level metrics can be calculated, depending upon the research hypotheses [23,24].

Physical and design characteristics data will be extracted directly from AutoCAD files and environmental surveys. Some physical and design characteristics (eg, distance from patient bed to patient bathroom) will be calculated within the AutoCAD program itself (Multimedia Appendix 4). Use of AutoCAD files, environmental surveys, and Depthmap reduces the need for extensive on-site measurement by hospital staff or researchers. This methodology ensures reliability of data collection across sites and minimizes measurement error that occurs by direct measurement.

#### Statistical Analysis

Statistical and graphic comparisons will be made between high-fall and low-fall units for the variables in Multimedia Appendix 4. The presence of statistically significant associations between hospital unit fall category (high, low) and each unit design measure will be tested separately as follows: a 2-sample *t* test for continuous measures (accessibility, visibility, corridor length) and a chi-square test for categorical measures (ie, single versus multioccupancy patient rooms).

### Ethical Approval and Consent to Participate

The research protocol was approved by the University of Florida and University of South Florida institutional review boards and VA Research Committees at both sites. Local union approval was obtained to conduct the walk-through interviews, and written informed consent was obtained before the interviews. VHA Labor Relations and the National Nurses Union reviewed the protocol and instruments and provided concurrence to conduct the nurse environmental assessment survey. The environmental assessment surveys were emailed; a waiver of documentation of informed consent was approved as the completion of the survey implied consent. Approved informed consent language was included on the first page of the environmental assessments.

## Results

All study approvals were obtained by January 2018. From January to March 2019, we conducted walk-through interviews and finalized the environmental assessment survey. By November 2019, we identified 25 units with higher-than-expected fall rates and 25 units with lower-than-expected rates. In January 2020, we began to request AutoCad files from these units. In January and August 2020, we surveyed facilities managers and nurse managers, respectively. We expect to complete data analysis by February 2022. Dissemination will occur during the following year.

## Discussion

### Principal Findings

Patient falls are the most common adverse events reported in hospitals [1,25]. Although it is well understood that the physical hospital environment is a common contributing factor to falls with injuries [26], there are significant gaps in our knowledge about the relationship between inpatient unit design and fall rates. The few studies that have examined unit design have been conducted in a single hospital or a small number of inpatient units, limiting generalizability [27,28]. Furthermore, no studies have focused on unit design and falls in VHA medical centers. Thus, the overarching goal of this study is to identify unit design factors contributing to inpatient falls within the VHA.

### Study Strengths

To our knowledge, this study represents the first attempt to study hospital falls in a large multihospital system. Hospitals employ numerous approaches to develop fall prevention policies. In general, the procedures are largely directed at nursing techniques and include identifying patients who are at high risk of falling and using clinical judgment to decide which of a myriad of strategies will reduce fall risk [29]. A quantitative review found no evidence of benefit among published nursing-oriented hospital fall prevention studies using concurrent controls (incidence rate ratio 0.92; 95% CI 0.65-1.30) [30,31]. Even though tremendous resources have been dedicated to reduce falls in VHA hospital units, a wide variation in fall rates still occurs. The advantages of the proposed research design are that it leverages the power of large VA administrative databases to identify nursing units that have the highest and lowest risk-adjusted fall rates and conducts in-depth analyses using physical measurements of environmental factors to identify factors contributing to fall rates. We will employ a novel analysis of floor plans using Depthmap spatial software to investigate unit design factors between the units with high and low fall rates. These space syntax measures could be used as physical environmental factors in future research examining a range of contextual factors—social, personal, organizational, and environmental—contributing to patient falls. In addition, the findings from this study can contribute to development and refinement of evidence-based design guidelines for hospitals and health care settings such as those of the Facility Guidelines Institute and the VA.

### Study Limitations

The walk-though portion of the study had 3 limitations we attempted to minimize through design of the study. First, the facilities selected for walk-through interviews formed a convenience sample, so they may not be representative of all VHA medical/surgical units. Given that the facilities available were built or renovated during different time periods, they provided for variations that may exist across the VHA. Second, because units are very busy places with high demands on staff, we included multiple staff so that if one was called away the walk throughs could be completed without them. Staff not in the walk throughs covered their absences as they were able. Third, because walk-through interviews included managers and frontline staff, we were aware of the potential for group dynamics being influenced by power differentials. The research team used validated focus group techniques to elicit feedback from all participant such as calling on individuals who were not as likely to offer responses as others.

Three limitations were noted for aim 2. First, each unit had only one participant per unit completing the environmental assessment survey, increasing the possibility of bias responses. To minimize this bias, we asked nurse managers, who are highly knowledgeable decision makers, to complete the surveys. Second, the risk adjustment models were complex and built on data from multiple data sources. While the VHA is an integrated health care system, we were faced with linking data that have no formal common identifying codes. All information available in each data source was used to match the units, and we conducted validity checks across datasets. Third, use of space syntax analysis requires some assumptions (eg, height of the agent line of sight). In this study, we considered that nurses are sitting in the nursing station and have a 360° view of their surroundings. However, they may be in the nursing station performing administrative tasks and not observing patients. Also, nurses are not always sitting in the nursing station; they might be walking in the corridor, which requires the assumption of standing height for the line of sight.

### Conclusions

To our knowledge, this study is the first to objectively identify spatial risks for falls in hospitals within a large multihospital system. Findings can contribute to evidence-based design guidelines for hospitals such as those of the Facility Guidelines Institute and the VA. The metrics for characterizing spatial features are quantitative indices that could be incorporated in larger scale contextual studies examining contributors to falls, which to date often exclude physical environmental factors at the unit level. Space syntax measures could be used as physical environmental factors in future research examining a range of contextual factors—social, personal, organizational, and environmental—that contribute to patient falls.

## Appendix

Multimedia Appendix 1 Walk-through interview guide.

Multimedia Appendix 2 Variables for creating models to identify fall rates that are higher and lower than expected.

Multimedia Appendix 3 Nurse environmental assessment survey.

Multimedia Appendix 4 Spatial and environmental variables.

## Abbreviations

AutoCAD: computer-aided design
VHA: Veterans Health Administration
VA: Department of Veterans Affairs
