# Supplementary material for: Examining the Relationship Between Environmental Factors and Inpatient Hospital Falls: Protocol for a Mixed Methods Study
Source: JMIR Res Protoc. 2021 Jul 13;10(7):e24974. doi: 10.2196/24974 (PMC8317036; doi:10.2196/24974)
Supplement: Multimedia Appendix 1 [file resprot_v10i7e24974_app1.pdf]

Walk-through Interview Guide

(Note: Guide to be modified based on concurrent analyses)

Map of hospital units to be visited

Pre-Interview Script:

Hello, my name is_____________________and I will be leading this walk-through interview today. I would also like to introduce the research team <participating study staff will state names and their roles in the walk-through interview>. Our research team is examining ways in which the VA can design units to better prevent patient falls. You have been identified as someone with first-hand perspectives in fall prevention at this hospital and we would like to hear your opinions about unit design and layout and how design factors might impact falls in your units.

Before we get started, <study team member> will review the informed consent form with you and answer any questions you may have about your participation in this study.

<Following completion of consenting, the qualitative lead will resume leading the interview.>

To facilitate your recollections and opinions about the design features, we'd like to walk through the unit with you as we ask you questions. People see things in different ways so there are no right or wrong answers to any of these questions. Please feel free to share your point of view.

I'd like to establish some ground rules to help this walk-through interview run smoothly.

-The interview will be audio recorded and we will photograph some design features that you point out to us. The photos we take will not have any people or personally

identifiable information in them.
-No information that can identify you will be used on notes or other written materials collected during the interview today and your feedback will be kept confidential.
-We ask that only one person speak at a time, and be allowed to finish his or her point before someone else speaks. We do not want to miss any of your comments.
-You do not have to answer every question, but we do want to hear from everyone.
-There are no right or wrong answers so please feel free to share any feedback you think might be relevant.

Does anyone have any questions about this interview or the project?

Thank you for taking the time to join us for this interview, we really appreciate your help. Let's begin!

<u>Interview Script:</u>

First, I want to start by asking each of you to introduce yourselves. Please tell us your first name and a little bit about your job, especially as it relates to fall prevention.

Now as we walk through the unit, I'd like you to focus on your hospital's unit layout and patient room design. As we walk and talk, please feel free to indicate specific spaces or features that figure into your responses.

1. What are your opinions about how the layout of your unit may contribute, positively or negatively, to patient falls?

Probes: a) the length, width and configuration of the corridor,
b) the position(s) of the nurses' station(s),
c) the placement of the patient rooms along the corridor,
d) or the placement of doors (and interior windows) to patient rooms off the corridor?

2. Could unit design have an impact on falls in your hospital? (Probe: Why or why not?)

3. Do you think unit design – that is, the space outside the patient rooms -- has an impact on how staff are able to view patients as they ambulate or get out of their beds? (Probe: Why or why not?)

Probes:

a) How could this be improved?
b) Location and orientation of nurses' stations
c) Number of nurses' stations,
d) Lighting and glare
e) Flooring in the corridor

4. What are your opinions about the patient rooms, including the bathrooms?

5. Could the patient room design have an impact on falls in your hospital? (Probe: What about -- lighting, door placement, courtesy drapes, relation and distance of patient bed to bathroom, noise, bathroom layout and grab bars, amount and placement of furniture and equipment?)

   How could this be improved?

6. As we walk through the unit, please describe examples of where patient falls have occurred, and whether you thought the layout of the unit or the patient room could have had an influence on that fall or its outcome?

7. Do you have any other ideas that don't necessarily involve the unit layout or patient room design?

Next, I'd like to hear your thoughts on some general aspects of fall prevention, whether related to unit design factors or not.

1. What do you think are the most important contributors to patient falls?
2. Are any of these preventable? (If yes…) Which ones?
3. What are some factors at *this hospital* that seem to influence the risk of inpatient falls?
4. What efforts are being made at the leadership level to change these factors?

Is there anything else you'd like to add that you might not have had a chance to say?

<u>*** Thank you so much for taking the time to share your opinions. We really appreciate your input!!</u>
