# Supplementary material for: Examining the Relationship Between Environmental Factors and Inpatient Hospital Falls: Protocol for a Mixed Methods Study
Source: JMIR Res Protoc. 2021 Jul 13;10(7):e24974. doi: 10.2196/24974 (PMC8317036; doi:10.2196/24974)
Supplement: Multimedia Appendix 2 [file resprot_v10i7e24974_app2.docx]

| Variables for creating models to identify higher- and lower-than-expected fall rates | | |
| --- | --- | --- |
| **Variable Name** | **Operational Definition** | **Source*** |
| Dependent Variable | | |
| Unit Fall Rate | Number of falls per 1000 bed days of care. Calculated as # falls/bed days*1000. | IPEC |
| Facility-Level Independent Variable | |  |
| Facility Complexity | Facility complexity level as defined by the Office of Productivity, Efficiency, and Staffing (OPES). The VHA Facility Complexity Model classifies VHA facilities at levels 1a, 1b, 1c, 2, or 3, with level 1a facilities being the most complex and level 3 the least complex. | VHA Support Services Center (VSSC) |
| Geographic Location | Regional designation of facility | VINCI CDW Managerial Cost Accounting – Ward File |
| Unit-Level Independent Variables | |  |
| Nursing Staffing | Total productive hours (normal + regular + comp time earned + overtime). | CDW - Managerial Cost Accounting – ABLCC file |
| Type of Unit | Units are identified as Acute Medicine, Acute Surgery, or Acute Medicine/Surgery. | IPEC |
| Patient-Level Independent Variables | |  |
| Age | Mean or median age of patients on unit. | VINCI CDW-Patient Domain |
| Gender | Mean bed days of care for males | VINCI CDW-Patient Domain |
| Severity of Illness/Co-morbidities | To estimate the impact of clinical co-morbidities on the risk of falling we use a “Nosos” a risk adjustment algorithm developed by Wagner et al. The Nosos scores are computed by first computing the CMS-VS21 risk scores using the V21 program. These risk scores, along with the additional factors, are then used as predictors in a regression model to model the annual VA cost for each patient. Estimates are rescaled so that mean Nosos scores for the population always equal one. [25] Mean bed days of care per month of patient in the 25^th^ percentile or lower and patients in the 75 percentile or higher. | VINCI CDW Health Economics Resource Center (HERC) datasets |
| Surgical Procedure | Flag indicating the presence of a surgical DRG. Operationalized as dichotomous variable: “yes” or “no” surgery during hospitalization. Mean bed days of care for surgery per month. | VINCI CDW–Inpatient Domain |
| Psychotropic Drugs | The presence of any of a list of anti-psychotic, anti-depressant, and sedative hypnotic drugs per VA formulary. Mean bed days of care per month. | VINCI CDW-Pharmacy BMCA Domain |
| * We will access most of the data from the Corporate Data Warehouse (CDW) through the VA Informatics and Computing Infrastructure (VINCI). Fall outcome data was accessed from Inpatient Evaluation Center (IPEC) directly through the national program office. All data will be housed in secure files on the VINCI workspace. | | |
