# Supplementary material for: Examining the Relationship Between Environmental Factors and Inpatient Hospital Falls: Protocol for a Mixed Methods Study
Source: JMIR Res Protoc. 2021 Jul 13;10(7):e24974. doi: 10.2196/24974 (PMC8317036; doi:10.2196/24974)
Supplement: Multimedia Appendix 3 [file resprot_v10i7e24974_app3.pdf]

Nurse Environmental Assessment Survey

The following map shows the floor plan of your unit. Use it as a reference for answering the questionnaire.

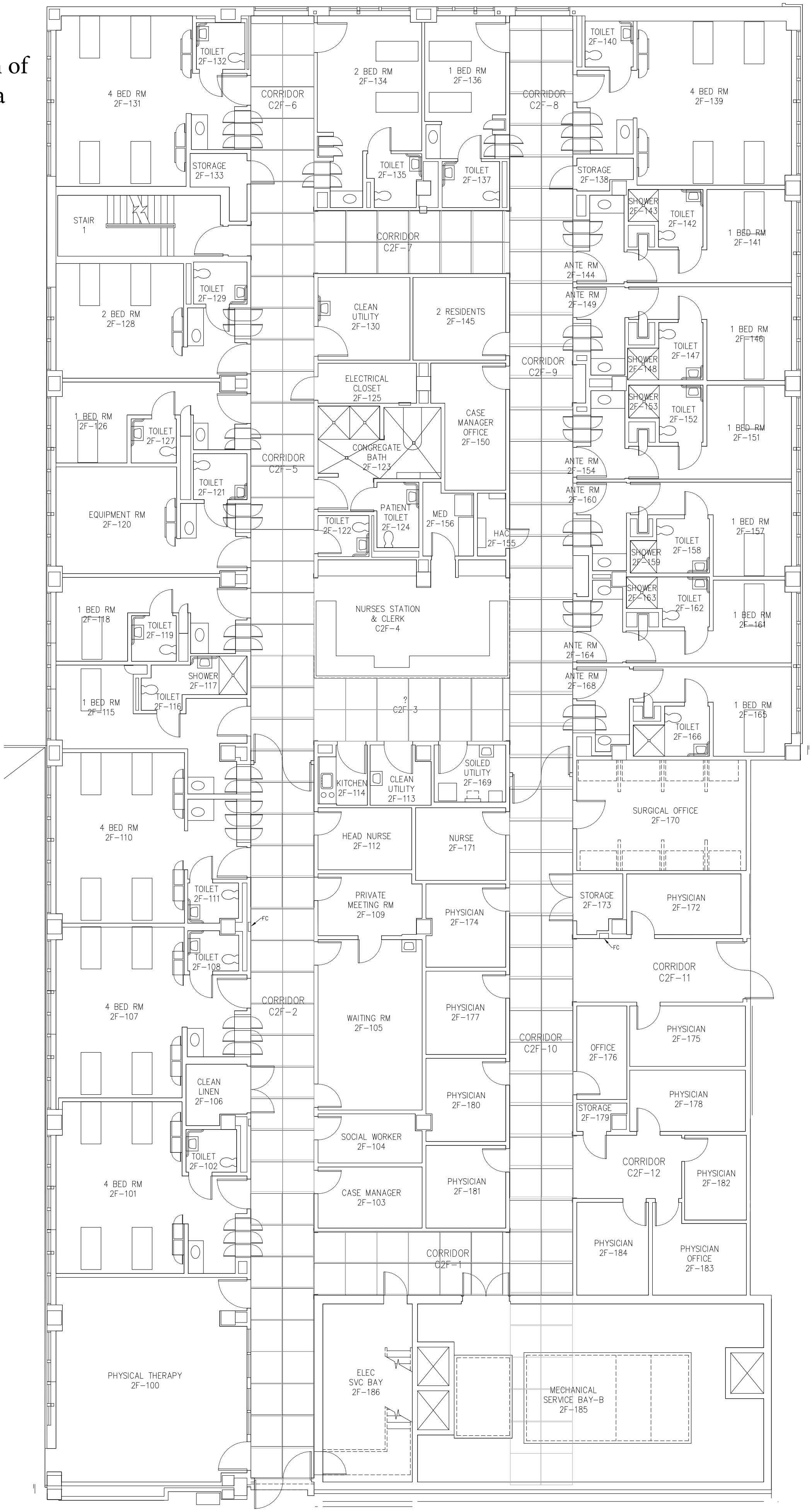

SECTION 1– UNIT CORRIDORS

1. If unit contains multiple nursing stations, on the unit map, please select which nursing station is considered the primary one for the unit.
2. **Does this unit have at least one Workstation on Wheels (WOW) or Computer on Wheels (COW)?**
   - ❑ Yes
   - ❑ No (Skip to Question 4)
3. **If #2 is YES, do nurses use them in the corridor on a regular basis?**
   - ❑ Yes
   - ❑ No
4. **Are any of the following types of equipment typically parked in the unit corridors?** (Examples: Automated medication dispersal cart, Stretchers, Wheelchairs, Walkers, and/or Laundry bins)
   - ❑ Yes
   - ❑ No
5. **Using the map, click on the corridor segments where equipment such as Medication carts, Stretchers, Wheelchairs, Walkers, Laundry bins, etc are typically parked** (e.g., stays there for at least a shift)**.**

SECTION 2 – PATIENT ROOMS

6. **On this unit, do you generally assign high risk for falls patients to certain rooms?**
   - ❑ Yes: (list the room number) ___________________________________
   - ❑ No: (list a room number representative of most rooms on this unit that you are using to complete the following questions) _____________________________

For Question 7, if there is a specific room that is **preferred for assigning patients** that are at high risk for falls, please answer using that room as an example. If there is not a preferred room, answer the questions using a room you consider representative of most rooms on this unit.

7. Please check the *physical characteristics of the patient room* that make the selected room *MORE* preferable to assign high fall risk patients.

Physical Characteristics	Comment on characteristics that make this room preferable for high fall risk patients.
❑ Location on the unit	
❑ Room layout	
❑ Patient bed location	
❑ Equipment in the room	
❑ Technologies	
❑ Pathway from patient bed to bathroom	
❑ Bathroom location	
❑ Bathroom door	
❑ Bathroom characteristics	
❑ Other physical characteristics not mentioned above	

For Questions 8 and 9, if there is a specific room that is **least preferred for assigning patients** that are at high risk for falls, please answer using that room as an example. If there is not a room like that on this unit, answer the questions using a room you consider representative of most rooms on this unit.

8. Is there a specific room that is least preferred for placing patients at high risk for falls?

❑ Yes (list the room number) ____________________

❑ No (list the room number that you are using to complete the following questions) _____ ________________________________

9. Please check all the *physical characteristics of the patient room* that make it *LEAST* preferred to assign high fall risk patients.

Physical Characteristics	Describe how these characteristics make this room less preferable for high fall risk patients.
❑ Location on the unit	
❑ Room layout	
❑ Patient bed location	
❑ Equipment in the room	
❑ Technologies	
❑ Pathway from patient bed to bathroom	
❑ Bathroom location	
❑ Bathroom characteristics	
❑ Bathroom door	
❑ Other physical characteristics not mentioned above	

SECTION 3 – PATIENTS ASSIGNMENTS & MONITORING

10. **Is it the primary practice on this unit that nurses should be assigned to patients in adjacent rooms or within close proximity to each other?**

- ❑ Yes, it is a primary practice
- ❑ It is occasionally practiced but not primarily
- ❑ No, we do not assign patients based on proximity

11. **What else about the design and physical environment on your unit do you believe affects patients' risk for falling?**

    ____

Submit Form

FACILITIES ENVIRONMENTAL ASSESSMENT SURVEY (FEAS)

Instructions: For each patient room on the unit, please enter the room number and the number of beds in each room. Use the legend to respond to the questions about visibility and flooring for each patient room and bathroom, where applicable. Once you complete the survey for the selected unit(s), please submit the survey online or email the survey to Slande.Alliance@va.gov.

Please list the room numbers for this unit and answer the questions about visibility into the patient rooms, as well as flooring types in the patient rooms and bathrooms.

LEGEND:

**ROOM INTERIOR**	**FLOORING**
**GD:** Glass or glazing in door<br>**WW:** Window in corridor wall	**CT:** Ceramic Tile<br>**PT:** Porcelain Tile<br>**RS:** Resinous Flooring<br>**VF**: Vinyl Flooring such as Luxury Vinyl Tile, Vinyl Composition Tile, or Welded Seams Vinyl<br>**N/A:** The patient room does not have a bathroom

Unit:

Room Number	Number of beds	Is the interior of the room visible when door is closed?	Flooring	
			Patient Room	Bathroom
		☐ Yes, GD ☐ Yes, WW ☐ No	☐VF ☐ Other	☐CT ☐PT ☐RS ☐VF ☐Other ☐N/A
		☐ Yes, GD ☐ Yes, WW ☐ No	☐VF ☐ Other	☐CT ☐PT ☐RS ☐VF ☐Other ☐N/A
		☐ Yes, GD ☐ Yes, WW ☐ No	☐VF ☐ Other	☐CT ☐PT ☐RS ☐VF ☐Other ☐N/A
		☐ Yes, GD ☐ Yes, WW ☐ No	☐VF ☐ Other	☐CT ☐PT ☐RS ☐VF ☐Other ☐N/A
		☐ Yes, GD ☐ Yes, WW ☐ No	☐VF ☐ Other	☐CT ☐PT ☐RS ☐VF ☐Other ☐N/A
		☐ Yes, GD ☐ Yes, WW ☐ No	☐VF ☐ Other	☐CT ☐PT ☐RS ☐VF ☐Other ☐N/A
		☐ Yes, GD ☐ Yes, WW ☐ No	☐VF ☐ Other	☐CT ☐PT ☐RS ☐VF ☐Other ☐N/A
		☐ Yes, GD ☐ Yes, WW ☐ No	☐VF ☐ Other	☐CT ☐PT ☐RS ☐VF ☐Other ☐N/A
		☐ Yes, GD ☐ Yes, WW ☐ No	☐VF ☐ Other	☐CT ☐PT ☐RS ☐VF ☐Other ☐N/A
		☐ Yes, GD ☐ Yes, WW ☐ No	☐VF ☐ Other	☐CT ☐PT ☐RS ☐VF ☐Other ☐N/A
		☐ Yes, GD ☐ Yes, WW ☐ No	☐VF ☐ Other	☐CT ☐PT ☐RS ☐VF ☐Other ☐N/A
		☐ Yes, GD ☐ Yes, WW ☐ No	☐VF ☐ Other	☐CT ☐PT ☐RS ☐VF ☐Other ☐N/A
		☐ Yes, GD ☐ Yes, WW ☐ No	☐VF ☐ Other	☐CT ☐PT ☐RS ☐VF ☐Other ☐N/A
		☐ Yes, GD ☐ Yes, WW ☐ No	☐VF ☐ Other	☐CT ☐PT ☐RS ☐VF ☐Other ☐N/A
		☐ Yes, GD ☐ Yes, WW ☐ No	☐VF ☐ Other	☐CT ☐PT ☐RS ☐VF ☐Other ☐N/A

FACILITIES ENVIRONMENTAL ASSESSMENT SURVEY (FEAS)

		☐ Yes, GD ☐ Yes, WW ☐ No	☐VF ☐ Other	☐CT ☐PT ☐RS ☐VF ☐Other ☐N/A
		☐ Yes, GD ☐ Yes, WW ☐ No	☐VF ☐ Other	☐CT ☐PT ☐RS ☐VF ☐Other ☐N/A
		☐ Yes, GD ☐ Yes, WW ☐ No	☐VF ☐ Other	☐CT ☐PT ☐RS ☐VF ☐Other ☐N/A
		☐ Yes, GD ☐ Yes, WW ☐ No	☐VF ☐ Other	☐CT ☐PT ☐RS ☐VF ☐Other ☐N/A
		☐ Yes, GD ☐ Yes, WW ☐ No	☐VF ☐ Other	☐CT ☐PT ☐RS ☐VF ☐Other ☐N/A
		☐ Yes, GD ☐ Yes, WW ☐ No	☐VF ☐ Other	☐CT ☐PT ☐RS ☐VF ☐Other ☐N/A
		☐ Yes, GD ☐ Yes, WW ☐ No	☐VF ☐ Other	☐CT ☐PT ☐RS ☐VF ☐Other ☐N/A
		☐ Yes, GD ☐ Yes, WW ☐ No	☐VF ☐ Other	☐CT ☐PT ☐RS ☐VF ☐Other ☐N/A
		☐ Yes, GD ☐ Yes, WW ☐ No	☐VF ☐ Other	☐CT ☐PT ☐RS ☐VF ☐Other ☐N/A
		☐ Yes, GD ☐ Yes, WW ☐ No	☐VF ☐ Other	☐CT ☐PT ☐RS ☐VF ☐Other ☐N/A
		☐ Yes, GD ☐ Yes, WW ☐ No	☐VF ☐ Other	☐CT ☐PT ☐RS ☐VF ☐Other ☐N/A
		☐ Yes, GD ☐ Yes, WW ☐ No	☐VF ☐ Other	☐CT ☐PT ☐RS ☐VF ☐Other ☐N/A
		☐ Yes, GD ☐ Yes, WW ☐ No	☐VF ☐ Other	☐CT ☐PT ☐RS ☐VF ☐Other ☐N/A
		☐ Yes, GD ☐ Yes, WW ☐ No	☐VF ☐ Other	☐CT ☐PT ☐RS ☐VF ☐Other ☐N/A
		☐ Yes, GD ☐ Yes, WW ☐ No	☐VF ☐ Other	☐CT ☐PT ☐RS ☐VF ☐Other ☐N/A

Submit Form
