# Supplementary material for: Examining the Relationship Between Environmental Factors and Inpatient Hospital Falls: Protocol for a Mixed Methods Study
Source: JMIR Res Protoc. 2021 Jul 13;10(7):e24974. doi: 10.2196/24974 (PMC8317036; doi:10.2196/24974)
Supplement: Multimedia Appendix 4 [file resprot_v10i7e24974_app4.docx]

|  |  | | |
| --- | --- | --- | --- |
| Spatial and Environmental Variables | | | |
| **Variable Name** | | **Definition** | **Source** |
| Overall Integration | | Space syntax measure of overall inter-visibility of the layout of the unit. | Depthmap |
| Patient room integration | | Space syntax measure of inter-visibility of the layout comprising only nursing stations and patients’ rooms | Depthmap |
| Visual Step Depth | | Space syntax measure that indicates the number of turns (plus one) needed to transverse from designated location (nurses’ stations) to other locations (patient rooms) within the plan. Every space that is directly visible from selected origin counted as one step away from that origin. It represents the degree of accessibility between points of interests. | Depthmap |
| Metric Step Depth | | Space syntax measure of the number of metric steps (walking distances) from each designated origin (nursing station) to all segments (patient rooms) in the unit. | Depthmap |
| Patient Room Occupancy | | % of unit with single occupancy patient room; multiple occupancy rooms | Survey, AutoCAD® |
| Nursing Stations | | Count of nursing stations on unit (and ratio of nursing stations to patient beds) | AutoCAD® |
| Corridor Path Length | | Linear feet of corridors along patient rooms and nurses’ stations; total linear feet of all corridors | AutoCAD® |
| Unit Layout Type | | Layout type based on configuration and relationship of corridor(s) and rooms (e.g. single corridor linear, double corridor linear, radial, etc.) | AutoCAD® |
| Patient Bathroom Location | | Location of patient bathroom in relation to patient beds (e.g. head of bed on same wall as bathroom door; head of bed on opposite wall, etc.) | AutoCAD® |
